# A new chemical entity dissociates amyloid‐β aggregation and alleviates Alzheimer‐induced cognitive decline

**DOI:** 10.1002/alz70859_100127

**Published:** 2025-12-25

**Authors:** JiMin Kim, Dohui Ku, InWook Park, Suhyun Ye, Illhwan Cho, Sunhee Lee, Ikyon Kim, YoungSoo Kim

**Affiliations:** ^1^ Yonsei Institute of Pharmaceutical Sciences, Incheon, Incheon Korea, Republic of (South); ^2^ Yonsei University, Incheon, Incheon Korea, Republic of (South); ^3^ Yonsei University, Yeonsu‐gu, Incheon Korea, Republic of (South); ^4^ Yonsei Institute of Pharmaceutical Sciences, Yeonsu‐gu, Incheon Korea, Republic of (South); ^5^ College of Pharmacy, Yonsei University, Incheon, Incheon Korea, Republic of (South)

## Abstract

**Background:**

Abnormal accumulation of amyloid‐β (Aβ) aggregates in the brain is a prominent pathology in Alzheimer’s disease (AD). This phenomenon is strongly associated with cognitive deficits resulting from neuroinflammation and synaptic dysfunction. Recent studies in AD therapeutics development have highlighted the prospect of reducing amyloid burden in treating AD patients as a promising treatment strategy, prompting the development of small molecule drugs capable of inducing chemical dissociation and clearance of Aβ.

**Method:**

Herein, we synthesized a chemical library of new chemical entities and selected a compound, IK‐9j, that demonstrated profound Aβ clearance efficacy via thioflavin T assays and dot blot analyses. The potential therapeutic efficacy of IK‐9j was evaluated utilizing an Aβ42‐infused mouse model.

**Result:**

The IK‐9j‐treated group exhibited significantly higher levels of short‐ and long‐term memory compared to the non‐treated group.

**Conclusion:**

Collectively, these results underscore the potential of IK‐9j as a novel small‐molecule therapeutic to mitigate Aβ‐associated cognitive deficits in AD.

